# Advances on cancer vaccine trials registered in China and the USA in 2014-2024

**DOI:** 10.3389/fonc.2025.1557253

**Published:** 2025-05-29

**Authors:** Wei Shi, Zhuo Chen, Qin Yu, Lingli Zhang

**Affiliations:** ^1^ National Drug Clinical Trial Institution of West China Second University Hospital, Sichuan University, Chengdu, China; ^2^ NMPA Key Laboratory for Technical Research on Drug Products In Vitro and In Vivo Correlation, West China Second Hospital, Sichuan University, Chengdu, China; ^3^ Children’s Medicine Key Laboratory of Sichuan Province, West China Second Hospital, Sichuan University, Chengdu, China; ^4^ Key Laboratory of Birth Defects and Related Diseases of Women and Children, Sichuan University, Ministry of Education, Chengdu, China; ^5^ Department of Pharmacy, West China Second University Hospital, Sichuan University, Chengdu, China; ^6^ Evidence-Based Pharmacy Center, West China Second University Hospital, Sichuan University, Chengdu, China; ^7^ Chinese Evidence-based Medicine Center, West China Hospital, Sichuan University, Chengdu, China

**Keywords:** cancer, vaccine, clinical trials, China, USA

## Abstract

**Objective:**

The clinical trials of cancer vaccine in China and the USA were summarized and compared over the past decade, thus putting forward suggestions for future clinical development in this field.

**Methods:**

Mainly based on the Centre for Drug Evaluation of China National Medical Products Administration website (NMPA) and ClinicalTrials.gov, a list and detailed information of cancer vaccine trials in China and the USA were acquired, respectively. Vaccines licensed for use in China and the USA were acquired from NMPA and Food and Drug Administration (FDA), respectively. Subgroup comparison in terms of initiated trials was conducted between the two countries.

**Results:**

In 2014-2024, there were 89 trials and 757 trials of cancer vaccine registered in China and the USA, respectively. In terms of the phase distribution, there were less phase I (28.1% vs 52.1%), phase II (15.7% vs 36.3%), and more phase III (40.5% vs 7.9%), phase IV (15.7% vs 3.7%) trials in China compared with those in the USA (χ^2^ = 116.58, P < 0.001). Regarding the trial scope, the proportion of global trials was significantly lower in China than that in the USA (2.2% vs 47.6%, χ^2^ = 66.78, P < 0.001). A significantly higher contribution rate was made by the top 20 pharmaceutical companies in China than that in the USA (16.9% vs 3.0%, χ^2^ = 35.43, P < 0.001). The numbers of trial status on open, completed and suspended in China are more than those in the USA (56.2%, 41.6%, 1.1% vs 43.6, 29.9%, 0.7%), while terminated trial is less than that in the USA (1.1% vs 9.1%). 5 cancer types were identified in China while there were more than 20 cancer types in the USA. Six cancer vaccines were approved in China while one was approved in the USA. With respect to indications, one cancer type was covered by the approved vaccines in China, which was fewer than the number of cancer types covered by the approved vaccines in the USA.

**Conclusion:**

Both China and the USA have made great progress in cancer vaccines in 2014-2024. The difference and gap between China and the USA highlight that more efforts should be paid on innovative cancer vaccines in China. Perhaps in the future, we can pay more attention to increasing the investment in drug research and development, carrying out more global multi-center clinical trials and participating more in trials led by top 20 pharmaceutical companies worldwide.

## Introduction

Cancer vaccine clinical trials are of great significance in the field of medicine (1, 2). In the late 19th century, an American doctor named William B. Coley found that some cancer patients’ tumors actually subsided after they were infected with erysipelas. And he concocted a bacterial mixture named “colitoxin” to treat cancer patients, which marked the beginning of cancer vaccine research. Provenge was approved by the Food and Drug Administration (FDA) in 2010 and became the world’s first approved therapeutic cancer vaccine. However, the vaccine company eventually went bankrupt because of the modest clinical efficacy and expensive price of the vaccine. In 2021, Patrick (3) revealed that the Neovax novel antigen vaccine, used to treat melanoma patients, could effectively control the immune response to tumor growth for up to four years. In 2023 (**Table 1**), a small mRNA pancreatic cancer vaccine trial jointly conducted by Germany and the USA achieved positive results, demonstrating the feasibility and potential of personalized cancer vaccines (4). In the same year, the investigational new drug (IND) of the first mRNA neoantigen vaccine named XH101 injection was accepted by the center for drug evaluation of National Medical Products Administration (NMPA) in China. In 2024, Geneos Therapeutics in the USA showed that the cancer vaccine Gnos-PV02 combined with PD-1 inhibitor has achieved encouraging efficacy in patients with advanced hepatocellular carcinoma. In 2024, the UK’s National Health Service (NHS) launched a pioneering clinical study of personalized cancer vaccines, which is the first large-scale trials of its kind in the world. These milestones not only demonstrate the great potential of cancer vaccines, but also provide new directions for future medical development.

**Table 1:** Key milestones in cancer vaccine clinical trials.

Year	Key milestones
2006	The FDA has approved the first HPV vaccine for the prevention of cervical cancer.
2023	A small mRNA pancreatic cancer vaccine trial conducted jointly by BioNTech in Germany and Memorial Sloan Kettering Cancer Center in New York achieved positive results.
2023	The IND of the first mRNA neoantigen vaccine named XH101 injection was accepted by the center for drug evaluation of NMPA in China.
2024	The NHS has launched a large-scale cancer vaccine trial programme that promises to improve survival and quality of life for cancer patients.
2024	Geneos Therapeutics in the USA announced that the cancer vaccine Gnos-PV02 combined with a PD-1 inhibitor has achieved encouraging efficacy and a good safety profile in patients with advanced hepatocellular carcinoma.

But cancer vaccine clinical trials also face some challenges (5) (1). Insufficient immunogenicity (4): Tumor antigens are often only slightly different from normal tissue antigens, so the degree of recognition and reaction of the immune system is relatively low (2). Neoantigen selection and prediction (6): Tumor cells at different sites may have different gene mutations and antigen expression, which make the selection of representative neoantigens a challenge (3). Delivery and targeting of vaccines: The current delivery system is inefficient in delivering antigens to the immune system to provoke an immune response (4). Validation and evaluation of clinical effects: Currently, there is a lack of uniform criteria for evaluating the clinical effectiveness of cancer vaccines (5). Economic and cost factors: High prices will limit the widespread use of cancer vaccines (6). Regulatory and ethical issues: Cancer vaccine trials require strict regulatory and more ethical considerations.

So, the purposes of this study were (1) to summarize and compare current progress of cancer vaccine clinical trials in China and the USA in 2014-2024, (2) to describe and compare new cancer vaccine approvals in China and the USA in 2014-2024, and (3) to put forward recommendations to further promote the development of cancer vaccines.

## Methods

### Data collection

Data for registered trials in China were acquired from the Centre for Drug Evaluation of China National Medical Products Administration website (NMPA). Data for registered trials in the USA were acquired from ClinicalTrials.gov. Data for licensed vaccines in China was acquired from NMPA, and data for licensed vaccines in the USA was from FDA. Cancer vaccine clinical trials data from 2024 are available until September 29, 2024.

### Inclusion and exclusion criteria

Inclusion criteria: We included clinical trials containing the word “cancer” and “vaccine” in any of the study title, drug names, conditions, or common test questions, etc.

Exclusion criteria were as follows: (1) We excluded clinical trials not in the time range of 2014-2024; (2) We excluded non-cancer vaccine trials; (3) We excluded trials whose trial status was NA.

### Statistical analysis

SPSS 26.0 software was used for all statistical analyses. Frequencies and percentages were used for the characteristic description of cancer vaccine clinical trials. The difference between groups was analyzed by Chi-square test using the number of cases or specific values. All statistical tests were two-sided, and the statistical significance was set to 0.05.

### Selection and general characteristics of studies

**Figure 1:**
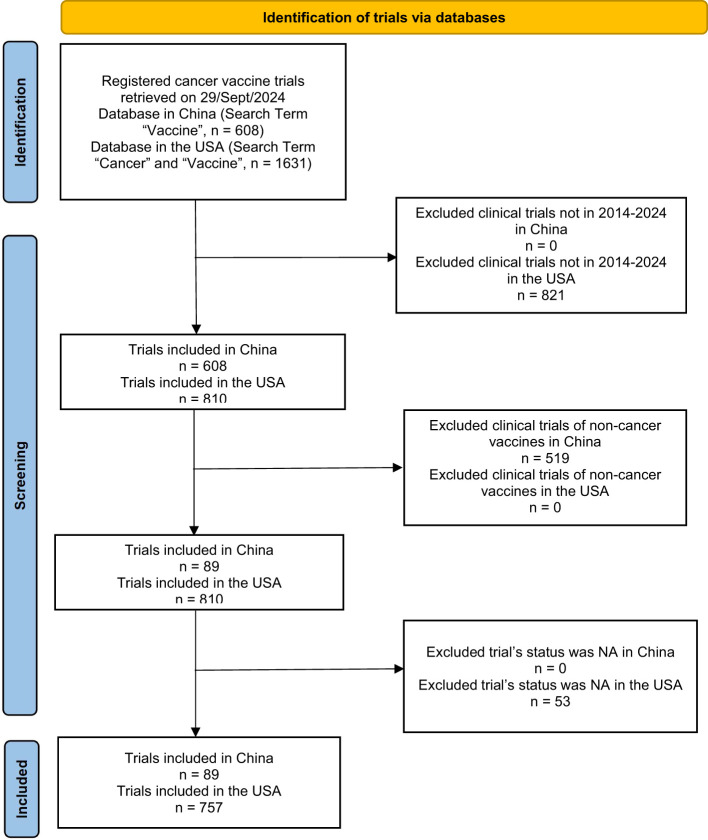
Flowchart for the selection and data sorting of cancer vaccine clinical trials registered in China and the USA in this study.

In the final analysis, 89 and 757 cancer vaccine clinical trials were included in China and the USA, respectively (**Figure 1**). A total of 519 non-cancer vaccine trials were excluded in China. A total of 874 trials were excluded in the USA, including trials that were not in the 2014-2024 time range (n = 821) and trials whose status was NA (n = 53).

## Results

In 2014-2024, there were 89 cancer vaccine trials registered in China, accounting for 0.3% of the total clinical trials of drugs in the country, while in the USA, a total of 757 cancer vaccine trials were identified, accounting for 0.02% of the total (**Table 2**). In terms of the phase distribution, there were less phase I (28.1% vs 52.1%), phase II (15.7% vs 36.3%), and more phase III trials (40.5% vs 7.9%), phase IV trials (15.7% vs 3.7%) in China compared with those in the USA (χ^2^ = 116.58, P < 0.001). Regarding the trial scope, 97.8% of the cancer vaccine trials in China were domestic, and the proportion of global trials was significantly lower than that in the USA (2.2% vs 47.6%, χ^2^ = 66.78, P < 0.001). As for the trial contribution by top 20 pharmaceutical companies, a significantly higher contribution rate was observed in China (16.9% vs 3.0%, χ^2^ = 35.43, P < 0.001).

**Table 2:** Cancer vaccine trials in China and the USA in 2014-2024.

Item	China		USA		Chi-square statistic	*P* value
	N	%	N	%		
Trial phase					116.58	<0.001
Phase I	25	28.1	394	52.1		
Phase II	14	15.7	275	36.3		
Phase III	36	40.5	60	7.9		
Phase IV	14	15.7	28	3.7		
Trial scope					66.78	<0.001
Global	2	2.2	360	47.6		
Domestic	87	97.8	397	52.4		
Top 20 pharmaceutical companies					35.43	<0.001
Yes	15	16.9	23	3.0		
No	74	83.1	734	97.0		

**Figure 2:**
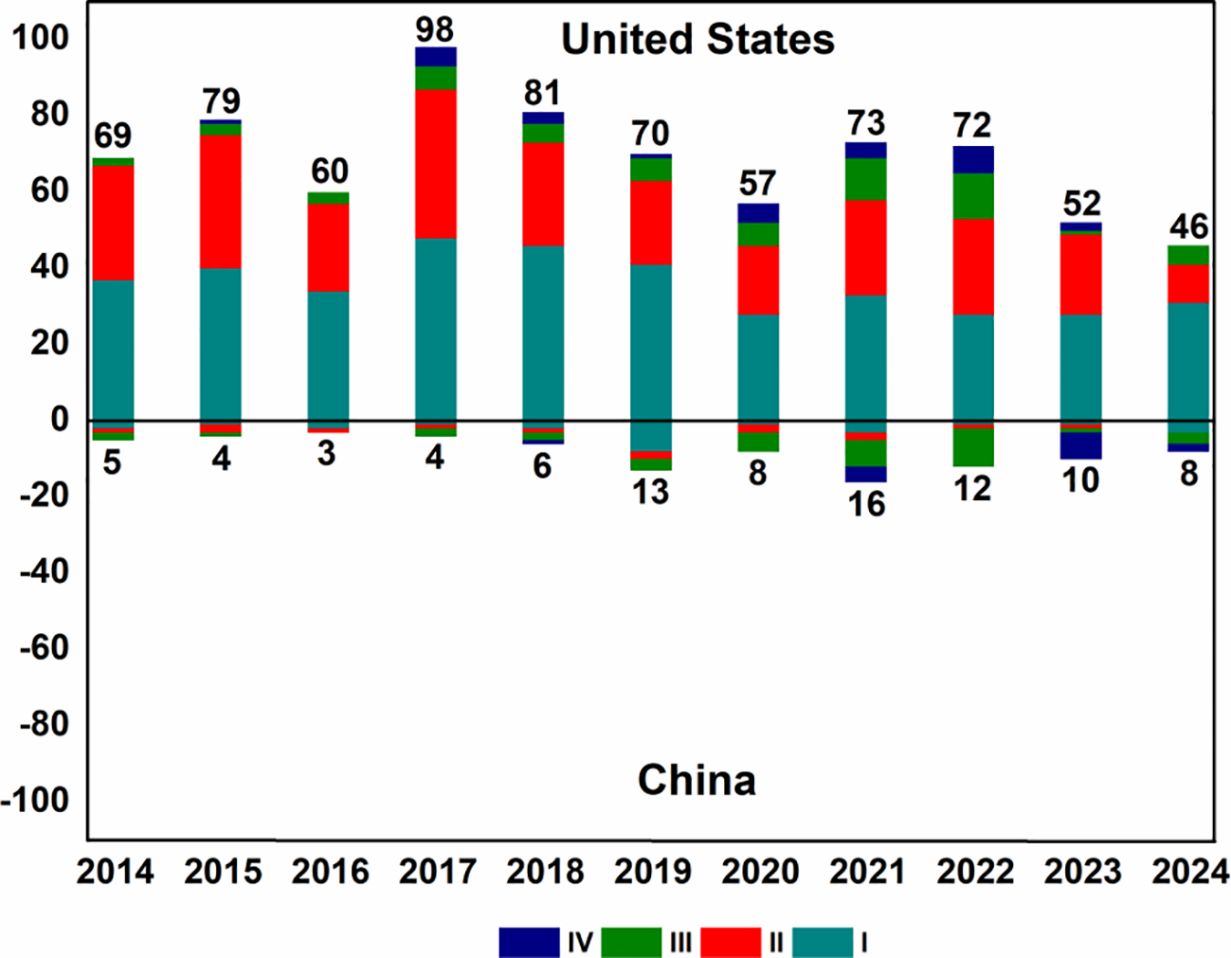
Analysis of numbers of cancer vaccine clinical trials by year and clinical stage in China and USA in 2014-2024.

The number of cancer vaccine clinical trials conducted by the USA ranges from 46 to 98 in the period from 2014 to 2024, with a peak in 2017 (98 trials) (**Figure 2**). In contrast, the number of cancer vaccine clinical trials conducted in China each year is relatively small, ranging from 3 to 16, with a peak in 2021 (16 trials).

**Figure 3:**
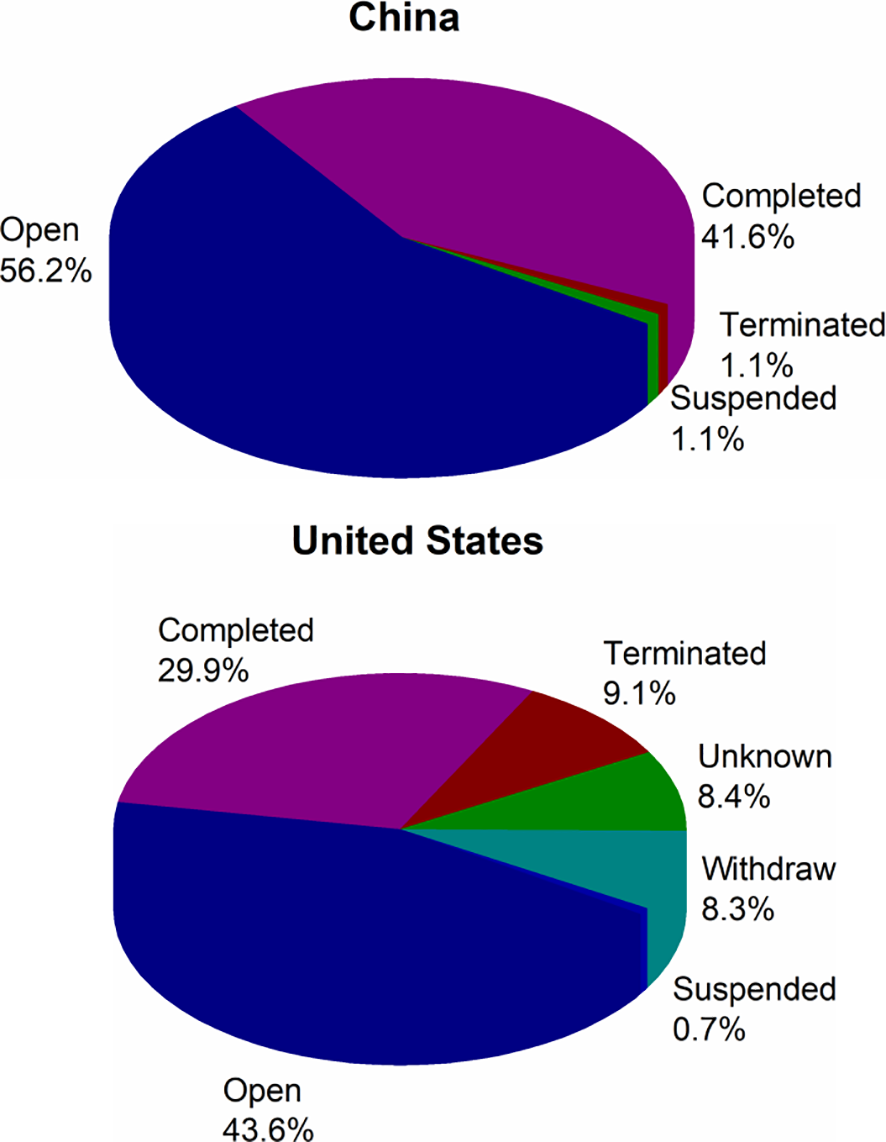
The trial status of cancer vaccine clinical trials in China and the USA.

Currently in China (**Figure 3**), 56.2% (50/89) of cancer vaccine trials are open, 41.6% (37/89) have been completed, and the remainder are either suspended (1.1%, 1/89) or terminated (1.1%, 1/89). And in the USA, 43.6% (330/757) of cancer vaccine trials are open, 29.9% (226/757) have been completed, 9.1% (69/757) are terminated, 8.4% (64/757) are unknown, and the remainder are either withdrawn (8.3%, 63/757) or suspended (0.7%, 5/757). The numbers of trials with the status of open, completed and suspended in China are more than those in the USA, while the number of trials with the status of terminated is less than that in the USA. There are two trial statuses in the USA, unknown and withdrawn, which do not exist in China.

**Figure 4:**
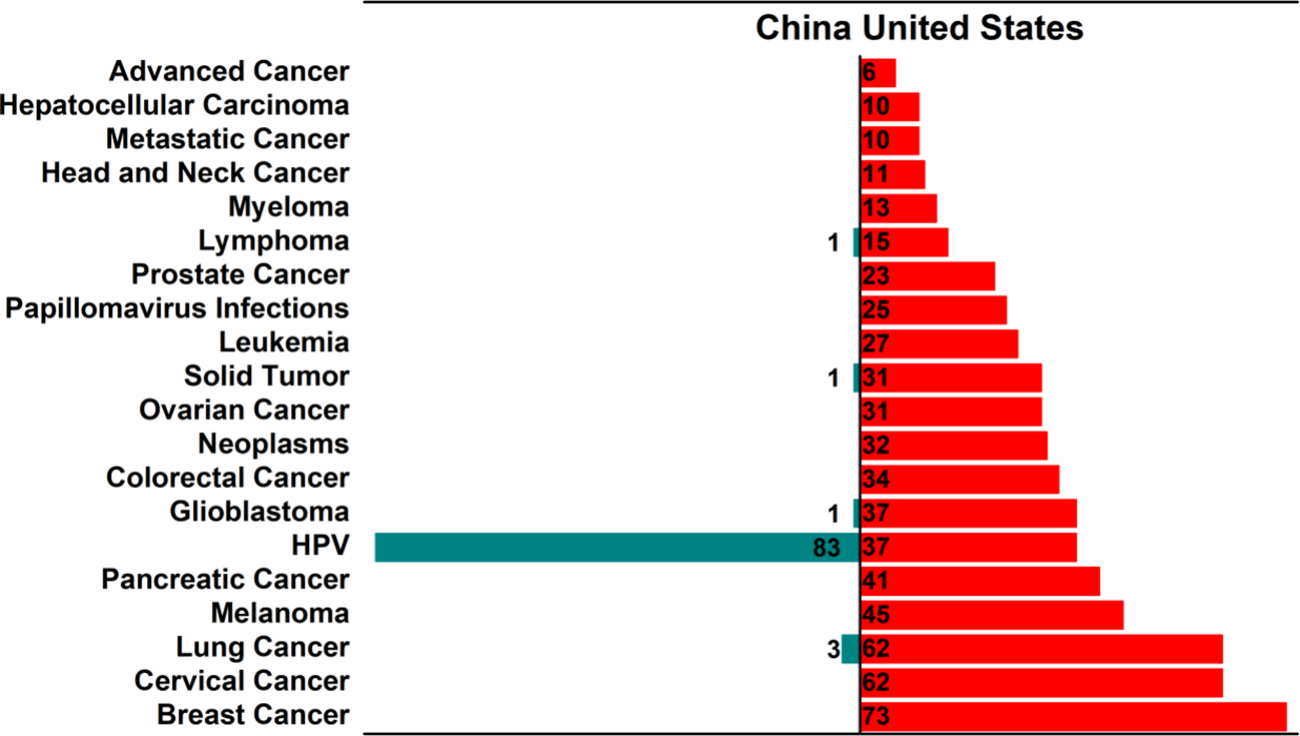
Top 20 cancer types of new vaccine trials in China and the USA in 2014-2024.

For cancer vaccine trials initiated in 2014-2024, 5 cancer types and more than 20 cancer types were involved in China and the USA, respectively (**Figure 4**). The five cancer types in the registered cancer vaccine clinical trials in China were as follows: human papilloma virus (HPV) (83, 93.3%), lung cancer (3, 3.4%), lymphoma (1, 1.1%), solid tumor (1, 1.1%), and glioblastoma (1, 1.1%), and this was different from the situation in the USA to some extent. Specifically, breast cancer (73, 9.6%), cervical cancer (62, 8.2%), lung cancer (62, 8.2%), melanoma (45, 5.9%), and pancreatic cancer (41, 5.4%) were the top 5 cancer types in the USA.

**Figure 5:**
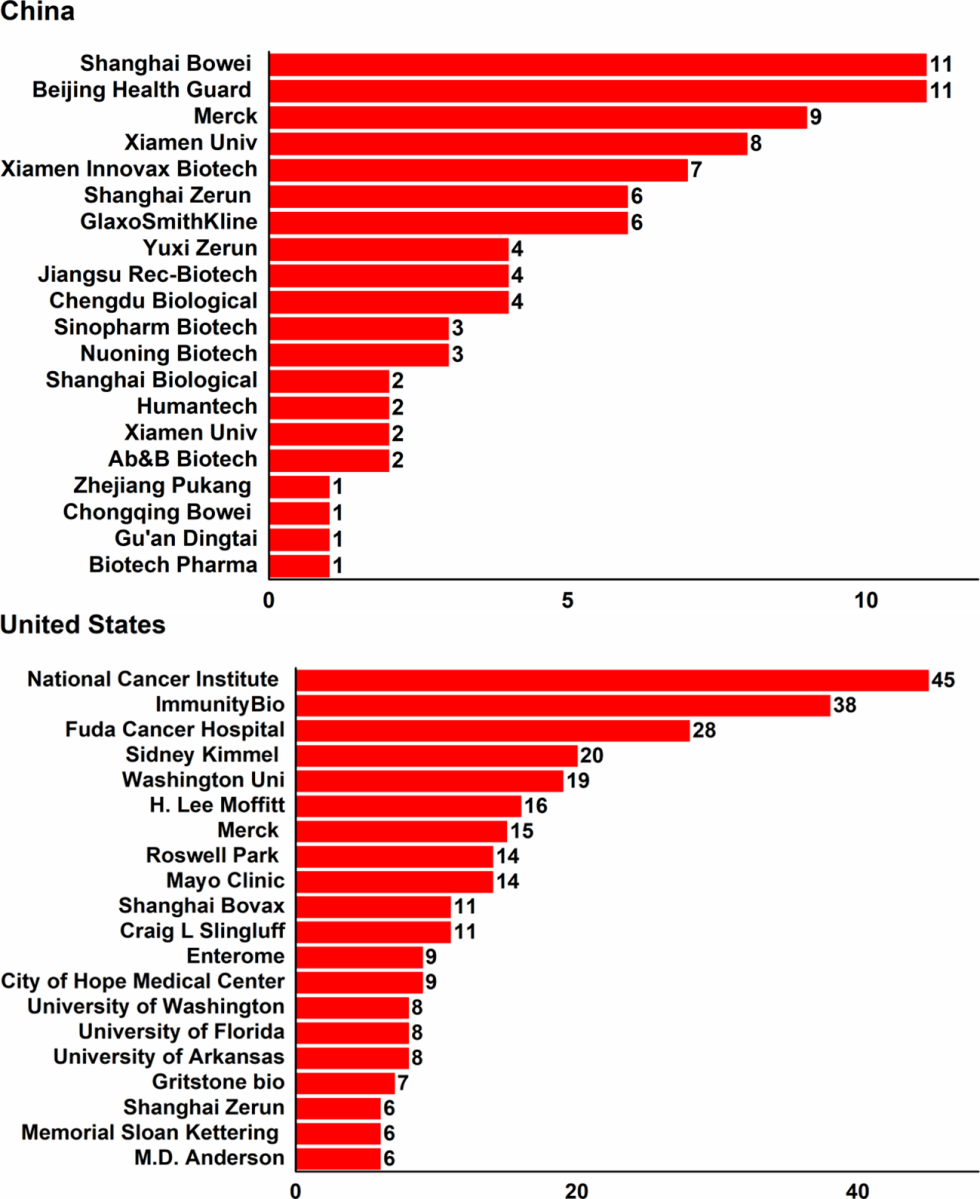
Top 20 sponsors of cancer vaccine trials in China and the USA in 2014-2024.

The top 20 sponsors of cancer vaccine trials were shown in **Figure 5**, who were responsible 98.9% and 39.4% of included trials in China and the USA, respectively. Among all sponsors, the USA had 5 belonging to the top 20 pharmaceutical companies worldwide, while China had 2. In detail, Shanghai Bowei and Beijing Health Guard initiated the largest number of cancer vaccine trials among Chinese sponsors in China, with the same total of 11 (12.4%), followed by Merck (9, 10.1%), Xiamen Univ (8, 9.0%), and Xiamen Innovax Biotech (7, 7.9%). And in the USA, National Cancer Institute initiated the most trials (45, 5.9%), followed by ImmunityBio (38, 5.0%), Fuda Cancer Hospital (28, 3.7%), Sidney Kimmel (20, 2.6%), and Washington Uni (19, 2.5%).

Six cancer vaccines were approved in China while one cancer vaccine were approved in the USA in 2014-2024 (**Table 3**, the cancer vaccines approved in China and the USA from 2006 to 2012 are only for historical review). In China, four vaccines were developed by Merck and two vaccines were developed by GlaxoSmithKline. While in the USA, one vaccine was developed by Merck. The cancer vaccines in both China and the USA were developed by top 20 pharmaceutical companies worldwide. Regarding the targeted cancer types, only cervical cancer was targeted by the vaccines in China, which was fewer than the targeted cancer types in the USA. Cervical cancer, vulvar cancer, vaginal cancer, anal cancer, oropharyngeal cancer and other head and neck cancers were the targeted cancer types in the USA. The tumor vaccines approved in China and the USA between 2014 and 2024 are all used for prevention and all adopt subunit vaccine platforms.

**Table 3:** Approval cancer vaccines in China and the USA.

No	Standard Tracking Number	Vaccine name	Pharmaceutical company	Top 20 pharmaceutical company worldwide	Indication	Approval year	Approval country	Purpose	Platforms
1	125126	Gardasil	Merck	Yes	Vulvar, vaginal cancer and Cervical cancer	2006	USA	Prevention	Subunit
2	JXSS0900012	Recombinant human papillomavirus (types 6,11,16,18) vaccine (yeast)	Merck	Yes	Cervical cancer caused by HPV types 6, 11, 16, 18	2009	China	Prevention	Subunit
3	JXSS0900013	Recombinant human papillomavirus (types 6,11,16,18) vaccine (yeast)	Merck	Yes	Cervical cancer caused by HPV types 6, 11, 16, 18	2009	China	Prevention	Subunit
4	BL 125259	Cervarix	GlaxoSmithKline	Yes	Cervical cancer and adenocarcinoma *in situ* caused by oncogenic HPV	2009	USA	Prevention	Subunit
5	102821	TICE BCG	Organon Teknika Corp belongs to Merck	Yes	Carcinoma in situ (CIS) of the urinary bladder	2010	USA	Therapeutic and prevention	Attenuated live vaccine platform
6	JXSS1200002	Human papillomavirus adsorbed vaccine	GlaxoSmithKline	Yes	Cervical cancer	2012	China	Prevention	Subunit
7	JXSS1200003	Human papillomavirus adsorbed vaccine	GlaxoSmithKline	Yes	Cervical cancer	2012	China	Prevention	Subunit
8	125508	Gardasil 9	Merck	Yes	Cervical, vulvar, vaginal, anal, oropharyngeal and other head and neck cancers caused by HPV	2014	USA	Prevention	Subunit
9	JXSS1800008	Nine-valent human papillomavirus vaccine (Saccharomyces Cerevisiae)	Merck	Yes	Cervical cancer	2018	China	Prevention	Subunit
10	JXSS1800009	Nine-valent human papillomavirus vaccine (Saccharomyces Cerevisiae)	Merck	Yes	Cervical cancer	2018	China	Prevention	Subunit
11	JXSS2101000	Nine-valent human papillomavirus vaccine (Saccharomyces Cerevisiae)	Merck (China)	Yes	Cervical cancer	2021	China	Prevention	Subunit
12	JXSS2101001	Nine-valent human papillomavirus vaccine (Saccharomyces Cerevisiae)	Merck (China)	Yes	Cervical cancer	2021	China	Prevention	Subunit
13	JXSS2100017	Bivalent human papillomavirus adsorbed vaccine	GlaxoSmithKline	Yes	Cervical cancer caused by HPV types 16 and 18	2021	China	Prevention	Subunit
14	JXSS2100018	Bivalent human papillomavirus adsorbed vaccine	GlaxoSmithKline	Yes	Cervical cancer caused by HPV types 16 and 18	2021	China	Prevention	Subunit

The cancer vaccines approved in China and the USA from 2006 to 2012 are only for historical review.

## Discussion

Cancer vaccine clinical trials have been developing rapidly in both China and the USA in recent years. The number of registered cancer vaccine trials has grown rapidly. Over the past decade there were 757 trials in the USA and 89 trials in China, respectively. Additionally, in terms of vaccine approvals, six cancer vaccines were approved in China while one were approved in the USA. There are several notable differences between China and the USA. Firstly, the proportion of global trials in China was significantly lower than that in the USA (2.2% vs 47.6%, χ^2^ = 66.78, P < 0.001). Secondly, the percentages of open and completed cancer vaccine trials in China were higher than those in the USA (56.2%, 41.6% vs 43.6%, 29.9%). Thirdly, the cancer types targeted by cancer vaccine trials were more diverse in the USA (more than 20 cancer types) than those in China (5 cancer types). What’s more, among all trial sponsors, the USA had 5 belonging to the top 20 pharmaceutical companies worldwide, while China had 2.

The number of registered cancer vaccine clinical trials in China lags behind that in the USA, and the variety of cancer types involved in these trials in China is more limited compared to the USA. It is speculated that the reasons may be as follows: (1) The pharmaceutical industry in the USA is developed with strong innovation ability, and there are constantly new drugs requiring clinical trials (7). Although China’s pharmaceutical industry has made rapid development in recent years, there is still a certain gap in innovative drug research and development compared with the USA (8). So the number of projects requiring clinical trials is relatively small. (2) In the USA, not only large hospitals, but also many small medical institutions and private clinics can participate in clinical trials (9). In China, according to relevant regulations (22), the medical institutions that can conduct clinical trials are mainly large medical institutions such as top-three hospitals, and the number is relatively small. (3) In some emerging disease fields in China, such as the fields of rare diseases and newly emerging infectious diseases, regulations may still be in the process of being improved, which restricts the implementation of relevant clinical trials to a certain extent. Perhaps in the future, we can increase the investment in drug research and development, improve the allocation of medical resources and perfect the related policies and regulations.

The proportion of global trials and the number of sponsorships from the world’s top 20 pharmaceutical companies were lower in China than those in the USA. Domestic pharmaceutical companies in China are facing greater pressure and challenges in the market competition, and they need to pay more attention to the demand of the domestic market (23). This can cause companies to have less incentive to conduct global clinical trials, preferring to conduct trials domestically to meet the needs of the domestic market. And the differences in regulations in different countries also increase the difficulty of multinational pharmaceutical companies to conduct clinical trials in China (10). Perhaps in the future, for China to fully integrate into the global cancer vaccine research and development system, domestic enterprises need to carry out more global multi-center clinical trials and participate more in trials led by top 20 pharmaceutical companies worldwide.

The number of cancer vaccines approved in China and the USA in the past decade is small. Maybe it is because the technical challenges of cancer vaccine development are high (24). When developing cancer vaccines, we need to consider not only the complexity of cancer and immune tolerance issues, but also the development of vaccine delivery systems (11). And its clinical trials are difficult and the costs of research and development are high. Technological breakthroughs bring new opportunities (12, 13). We believe the continuous optimization of mRNA technology, the development of novel adjuvants and multimodal vaccines all bring new opportunities for the development of cancer vaccines.

All the tumor vaccines approved in China and the USA from 2014 to 2024 serve a preventive purpose and utilize subunit vaccine platforms. Preventive tumor vaccines have achieved certain results in the prevention of some cancers (14–17). For example, the HPV vaccine has been effective in preventing cervical cancer. On the other hand, therapeutic tumor vaccines are still in the stage of continuous research and development as well as clinical trials (25). Although some vaccines have demonstrated a certain degree of efficacy in specific types of tumors, such as the therapeutic effect of TICE BCG in Carcinoma *in situ* (CIS) of the urinary bladder, further research and improvement are still needed before they can be widely applied in clinical practice (26). In the past few years, several new vaccine platforms have emerged in the field of cancer vaccines, such as subunit, adenovirus, mRNA, DNA, or others (27–29). Among them, the subunit vaccine platform has several notable characteristics (30). Firstly, it is extremely safe as it only contains purified antigenic components rather than the whole pathogen. Secondly, subunit vaccines are relatively stable and are easy to produce and store. Moreover, subunit vaccines can be designed to target specific antigens, thereby enhancing the specificity of the immune response. This makes them a very promising option in the research and development of tumor vaccines.

Furthermore, the majority of the cancer vaccine trials are focused on HPV diseases. We speculate that there are several reasons. (1) HPV has a high infection rate and carcinogenicity. Two high-risk types of HPV, HPV16 and HPV18, are responsible for more than 70% of cervical precancerous lesions and advanced cancers (14). (2) The development of HPV vaccines is feasible. The major structural proteins of HPV can self-assemble into immunogenic virus-like particles (VLPs), which resemble the natural virus but do not contain viral genes, and thus are non-infectious. Existing HPV vaccines, such as Gardasil and Cervarix, have been developed based on this principle. They have been proven to be safe and effective in preventing HPV infections and related precancerous lesions (15). (3) Developing HPV vaccines has significant social and economic benefits. Cervical cancer is a common gynecological malignant tumor and also a major public health issue (16). It has been found that the incidence rate of cervical cancer among women who have received the HPV vaccine has significantly decreased by 63%, and for women who were vaccinated before the age of 17, the decline in the incidence rate is as high as 88% (17). This will have a significant impact on reducing the global burden of cervical cancer and improving the quality of life of women, and will bring about remarkable social and economic benefits (18, 19).

However, China and the USA have still made good progress in the field of cancer vaccines. Chinese research teams continue to conduct in-depth research in tumor immunotherapy, providing theoretical support for the research and development of cancer vaccines. Zeng (20) systematically studied the molecular mechanism and targeted intervention of Epstein-Barr virus, which are of great significance. The “perivaccine” immune pre-stimulation strategy proposed by the team of Zhao and Nie can enhance the effect of subsequent tumor vaccine immunotherapy (21). In 2024, the mRNA tumor vaccine named WGC-043 injection, independently developed by Chengdu Wissin BioTech, was approved by both China (clinical trial number: CTR20243885) and the USA (clinical trial number: NCT05714748) to carry out phase I clinical trials. It provides a new therapeutic hope for patients with advanced Epstein-Barr virus positive solid tumor and hematological malignancy. In addition, the USA has made significant progress in the development of several vaccines in recent years, such as mRNA-4157 (v940) (clinical trial number: NCT05933577) and Gnos-pv02 (clinical trial number: NCT04251117). With the continuous development of cancer vaccine technology and the expansion of clinical applications, the market size of cancer vaccines will continue to expand, and international cooperation will become closer.

There may be some limitations in our study. In China, only cancer vaccine trials for registration purposes were included while investigator-initiated trials were not involved due to limited data availability. And we just made an overall description of cancer vaccine trials from a macro statistical point of view, so there might be a lack of in-depth focus.

## Conclusion

In conclusion, both China and the USA have made great progress in cancer vaccines in 2014-2024. Cancer vaccine clinical trials are an area of research full of challenges and opportunities. The gap compared with the USA in cancer vaccine trials highlights that more efforts should be paid to innovative agents and cancers unique to Chinese populations, as well as to facilitate global synchronous research and development in China. Perhaps in the future, we can pay more attention to increasing the investment in drug research and development, carrying out more global multi-center clinical trials and participating more in trials led by top 20 pharmaceutical companies worldwide. Through continuous efforts and innovation, it is expected to bring new therapeutic hope to many patients.

## Data Availability

The raw data supporting the conclusions of this article will be made available by the authors, without undue reservation.
